# The relationship between perceived functional difficulties and the ability to live well with mild‐to‐moderate dementia: Findings from the IDEAL programme

**DOI:** 10.1002/gps.5128

**Published:** 2019-05-20

**Authors:** Anthony Martyr, Sharon M. Nelis, Catherine Quinn, Jennifer M. Rusted, Robin G. Morris, Linda Clare, on behalf of the IDEAL programme team

**Affiliations:** ^1^ Centre for Research in Ageing and Cognitive Health, School of Psychology University of Exeter Medical School and College of Life and Environmental Sciences, St Luke's Campus Exeter UK; ^2^ Centre of Applied Dementia Studies University of Bradford Bradford UK; ^3^ School of Psychology University of Sussex Brighton UK; ^4^ Department of Psychology Institute of Psychiatry, Psychology and Neuroscience, King's College London London UK; ^5^ Wellcome Centre for Cultures and Environments of Health University of Exeter Exeter UK

**Keywords:** activities of daily living, Alzheimer's disease, carer stress, depression, quality of life

## Abstract

**Objectives:**

The objectives of the study are to investigate how different levels of functional ability relate to quality of life, well‐being, and satisfaction with life, conceptualised as reflecting capability to “live well” in people with dementia.

**Methods/design:**

Participants were 1496 people with mild‐to‐moderate dementia and 1188 informants who completed baseline assessments in the Improving the experience of Dementia and Enhancing Active Life (IDEAL) cohort study. Total self‐rated and informant‐rated scores on the Functional Activities Questionnaire were split into six ability levels to monitor how poorer functioning impacts the ability to live well. We also investigated the potential influence of sociodemographic and diagnostic variables, depression, cognition, and carer stress.

**Results:**

Multivariate multiple regression models found that people with dementia who had the greatest functional impairment according to self‐ratings and informant ratings had poorer living well scores than those with the least functional impairment. Sociodemographic and diagnostic factors and cognition had little impact on effect sizes. For self‐ratings, depression attenuated the relationship between functional ability and living well, whereas carer stress attenuated informant ratings.

**Conclusions:**

People with dementia with the least functional impairments had greater capability to live well than those with the most functional impairment. Even subtle perceived difficulties in functional ability had a detrimental effect on the ability of people with dementia to live well. Depression in people with dementia and carer stress in informants influenced these associations, and therefore, these factors should be routinely included in future research studies and clinical assessments.

**Key points**

- For people with dementia, there is a generally linear relationship between those with the least perceived functional impairments and those with the greatest perceived functional impairment and their ability to live well.
- Even subtle difficulties in activities of daily living have a detrimental effect on the ability of people with dementia to live well.
- Depression in people with dementia and carer stress in informants need to be routinely considered in future research studies and clinical assessments.

## 1 INTRODUCTION

Dementia refers to a group of progressive brain disorders associated with largely generalised mental functioning difficulties, including memory and other aspects of cognition, behavioural disturbances, and reduced activities of daily living (ADL).^1^, ^2^ Impairment in ADL is diagnostically central to dementia.^3^, ^4^ There is a hierarchical sequence of decline in ADL that distinguishes between basic ADL and instrumental ADL.^5^ In early‐stage dementia, basic ADL, such as bathing, eating, and dressing, show little decline,^6^ whereas instrumental ADL, such as using a telephone, managing finances, and managing medication, show noticeable decline,^7^, ^8^ and evidence suggests linkage between declining instrumental ADL and cognitive abilities.^9^, ^10^ This evidence led to the dependence framework, conceptualised as the measurable impact that concomitant cognitive and functional difficulties and increased neuropsychiatric symptoms exert as dementia severity increases.^11^

The dependence framework and the “disablement process” theory propose that increased dependence negatively affects quality of life (QoL).^11^, ^12^ People with dementia identify functional independence as an important factor in enhancing their QoL.^13^, ^14^, ^15^ In the United Kingdom, the Alzheimer's Society identifies improving the QoL of people with dementia by promoting functional ability and independence as one of its five priority goals for dementia care research.^16^ Recent dementia policy has prioritised enabling people to “live well” with dementia by improving health care and the quality of support offered to people with dementia.^17^, ^18^

A primary goal for dementia care is to maximise the ability to live well with the condition.^19^ Living well in people living with chronic illness and disability is defined as the best achievable health state that encompasses all dimensions of physical, mental, and social well‐being.^20^, ^21^, ^22^ While the concept of QoL concerns integration of interpersonal and health factors, as indicated above, the well‐being concept can be considered as a state of equilibrium or balance, which is affected by life events or challenges,^23^ while satisfaction with life entails a global evaluation of one's current life.^24^ Integration of these three concepts captures a more complete understanding of what it is to live well with a particular condition.^22^, ^25^, ^26^ Understanding the relationship between functional ability and subjective perceptions of living well is important, particularly as no preventive or curative treatment for dementia is currently available.

In people with dementia, reduced functional ability is consistently associated with higher depression, increased age, lower education, and poorer cognitive status.^2^, ^11^, ^27^, ^28^, ^29^, ^30^, ^31^, ^32^ Functional ability has also been investigated concerning self‐rated and informant‐rated QoL in dementia, with functional difficulties leading to poorer QoL outcomes.^33^, ^34^, ^35^, ^36^, ^37^, ^38^, ^39^ Investigating QoL in people with mild‐to‐moderate dementia typically involves use of either self‐ratings or informant ratings,^40^ and it is generally accepted that people with mild‐to‐moderate dementia can provide valid self‐ratings.^39^ In contrast, for assessing functional ability, informant ratings are the primary method of assessment,^2^, ^41^ often assumed to be the more accurate, with cognitive decline precluding the possibility of obtaining valid self‐ratings.^28^, ^42^, ^43^, ^44^ However, higher carer stress is consistently associated with lower informant‐rated functional ability^45^, ^46^ whereas carer stress is typically unrelated to objectively assessed functional performance.^32^, ^46^ We recently found that, when comparing self‐ratings and informant ratings of functional ability with objective performance, people with mild‐to‐moderate dementia were able to more accurately appraise their functional ability than their informants, with the latter typically underestimating ability.^32^ In view of this finding, it is important to consider how subjective perceptions of functional ability relate to both self‐rated and informant‐rated living well scores.

In summary, it is timely to investigate how different levels of functional ability relate to living well in people with mild‐to‐moderate dementia, as this could influence the type of care and support required and the timing of appropriate interventions. The present study has two aims. Firstly, since there is evidence that people with mild‐to‐moderate dementia can provide accurate self‐ratings of functional ability, we consider how different levels of self‐rated ability and informant‐rated ability relate to three indicators of living well: perceptions of QoL, satisfaction with life, and well‐being. Secondly, we aim to identify whether the level of functional ability across the spectrum of impairment affects subjective perceptions of living well, controlling for relevant variables including depression, cognition, and carer stress.

## 2 METHODS

### 2.1 Design

The Improving the experience of Dementia and Enhancing Active Life (IDEAL) programme is a 9‐year longitudinal cohort study investigating influences on living well with dementia.^25^, ^26^ This paper presents cross‐sectional data from version 2.0 of the IDEAL dataset for initial assessments conducted between July 2014 and August 2016. The cohort at baseline included 1547 participants with dementia together with 1283 informants, mostly spouses/partners. Only those with complete data for the Functional Activities Questionnaire (FAQ)^30^, ^47^ were included in the present analysis, yielding a sample of 1493 people with dementia and 1188 informants. Participants were recruited through UK National Health Service research networks across England, Scotland, and Wales. To be included, participants had to have a clinical diagnosis of dementia as judged by clinicians at recruitment sites and a score of 15 or above on the Mini‐Mental State Examination,^48^ indicating mild‐to‐moderate dementia, and the ability to communicate verbally in English. Exclusion criteria were comorbid terminal illness, inability to provide informed consent, and any known potential for home visits to pose a significant risk to researchers. Informants were recruited into the study if they were willing to take part and were proving regular care to the person with dementia.^49^ Full criteria for exclusion and consent are provided in the protocol.^25^ The IDEAL study was approved by the Wales Research Ethics Committee 5 (reference 13/WA/0405) and the Ethics Committee of the School of Psychology, Bangor University (reference 2014‐11684), and is registered with UKCRN, registration number 16593.

### 2.2 Measures

#### 2.2.1 Functional ability

To measure instrumental ADL, we employed an 11‐item FAQ, modified from the original 10 items to include a question concerning telephone use.^30^ Each item was rated on a 0 to 3 scale leading to a score range of 0 to 33; a higher score indicated greater perceived difficulty with functional ability. Both self‐rated (FAQ‐S) and informant‐rated (FAQ‐I) versions were used in the study. FAQ scores were split into six levels of impairment. Level 1 comprised those with no reported functional impairment (FAQ 0). To indicate impairment, a cut‐off score of 5 has been proposed.^47^ We have previously found that this cut‐off is commensurate with the amended FAQ scoring, and in our previous study, no one was reclassified as impaired using the self‐rated or informant‐rated amended FAQ.^30^ Therefore, level 2 included those who scored 1 to 5. An alternative FAQ cut‐off score of 9 has been recommended as indicative of impairment^44^ and is used primarily in North America;^50^ thus, level 3 included those who scored 6 to 9. The remaining three groups reflect equal score ranges comprising 8 points each: level 4 (10‐17), level 5 (18‐25), and level 6 (26‐33). Participants with dementia who had no informant participating in the study were included in the analyses using FAQ‐S ratings but not in the analyses using FAQ‐I ratings.

#### 2.2.2 Living well

We used three measures to assess living well with dementia. These were the QoL in Alzheimer's Disease scale score (QoL‐AD),^51^ Satisfaction with Life Scale score (SwLS),^52^ and the World Health Organization‐Five Well‐being Index (WHO‐5)^53^, ^54^ percentage score. For each measure, a higher score indicates greater capability to live well: QoL‐AD range 13 to 52, SwLS range 5 to 35, and WHO‐5 range 0 to 100. Both self‐rated and informant‐rated versions of each measure were used. For convenience, the three measures together will be referred to as “living well measures.”

#### 2.2.3 Cognition, mood, and carer stress

The following additional measures from the IDEAL dataset were included in this analysis. The Addenbrooke's Cognitive Examination‐III (ACE‐III)^55^ was used to measure cognition in participants with dementia. The ACE‐III is scored out of 100 with higher scores indicating better cognitive functioning. The Geriatric Depression Scale‐10 (GDS‐10)^56^ was used to measure depression in participants with dementia, with higher scores indicating more self‐rated depressive symptoms. For the purposes of the analysis, the sample was split into two groups: not depressed (GDS‐10 = 0‐3) and depressed (GDS‐10 = 4‐10). The Relatives' Stress Scale (RSS)^57^ measured the level of self‐reported carer stress; possible scores range from 0 to 60 with higher scores indicating greater carer stress.

#### 2.2.4 Sociodemographic and diagnostic variables

For the participants with dementia, we obtained information about age, gender, education, diagnosis, and relationship to the informant. Participants were classified into five groups based on age (younger than 65, 65‐69, 70‐74, 75‐79, and 80 years and older) and education (no qualifications, school leaving certificate at age 16, school leaving certificate at age 18, and university). Relationship to the informant was classified into three groups (spouse/partner, other, and no informant participating).

### 2.3 Procedure

Information was collected from people with dementia and informants who were visited at home by a researcher on three occasions spread over a few weeks. Informed consent was obtained from both the person with dementia and the informant (where available).

### 2.4 Planned analyses

A series of multivariate multiple regression analyses examined the relationship between the six FAQ impairment levels and scores on the living well measures, with separate analyses conducted for self‐ratings by the person with dementia and informant ratings. As the group with FAQ scores between 10 and 17 contained the second largest number of responses for both self‐rated and informant‐rated FAQ, it was used as the reference group in the regression analyses. Self‐rated living well measures were included in the self‐rated FAQ regression model, and informant‐rated living well measures were included in the informant‐rated FAQ model. For the first research aim, regression analyses were used to examine the unadjusted association between FAQ scores and living well measures for both self‐rated and informant‐rated scores. Further analyses were conducted adjusting for known covariates such as age, sex, education, and diagnosis. Further adjustments added ACE‐III and depression in the person with dementia (GDS‐10) to the model. A fifth model was tested with informant‐rated FAQ scores only, adding carer stress (RSS) to the model. Preprocessing of data included checking for normalised data assumptions for both individual measures and combined scores. All assumptions were met at each stage.

For the second research aim, as the three living well measures employed different scoring systems, scores were standardised. Multivariate multiple regression models for each rating type were repeated using the standardised scores so that the living well measures could be directly compared.

Multiple imputation was conducted to account for missing data. Ordinal variables were imputed using ordinal regression, and categorical variables were imputed using multinomial regression. The imputed model included all variables in the analysis. Estimates from 10 imputed datasets were combined using Rubin's rules.^58^

## 3 RESULTS

The 1493 participants with dementia were in the mild‐to‐moderate stages. There were more men than women with dementia, and Alzheimer's disease was the most frequent diagnosis (Table 1). The 1188 informants were mostly spouses, and there were more female than male informants. RSS scores indicate relatively mild levels of carer stress. Means for the three living well measures indicate that people with dementia rate their ability to live well more positively than informants; see Table S1 for mean and percentage data. The inclusion of the additional telephone use item in the amended version of the FAQ had little effect on the number of people classified as impaired; for self‐rated and informant‐rated FAQ, there were, respectively, five (0.3%) and six (0.5%) additional participants classified as impaired following inclusion of the additional item. There were slightly more classified as impaired with the amended FAQ using the cut‐off score of 9 with 22 (1.5%) and 17 (1.4%) for self‐rated and informant‐rated FAQ, respectively.

**Table 1:** Self‐ and informant‐rated functional ability across sociodemographic, diagnostic, and depression groups

	n (%)	FAQ‐S Mean (SD)	n (%)	FAQ‐I Mean (SD)
Diagnosis				
Alzheimer's disease	829 (55.5)	8.82 (7.36)	664 (55.9)	17.30 (8.35)
Vascular dementia	161 (10.8)	10.83 (8.21)	127 (10.7)	17.82 (8.43)
Mixed Alzheimer's and vascular dementia	317 (21.2)	9.73 (7.81)	242 (20.4)	18.00 (9.22)
Frontotemporal dementia	52 (3.5)	8.60 (7.02)	44 (3.7)	17.68 (9.43)
Parkinson's disease dementia	42 (2.8)	13.12 (7.94)	40 (3.4)	19.83 (8.27)
Dementia with Lewy bodies	51 (3.4)	14.59 (7.43)	41 (3.5)	22.00 (6.79)
Unspecified dementia/other	41 (2.7)	10.39 (8.59)	30 (2.5)	20.57 (9.52)
Education				
No qualifications	406 (27.2)	10.64 (7.56)	304 (25.6)	17.71 (8.28)
School leaving certificate at age 16	257 (17.2)	9.79 (7.78)	211 (17.8)	18.33 (8.42)
School leaving certificate at age 18	503 (33.7)	9.25 (7.77)	410 (34.5)	17.90 (8.80)
University	295 (19.8)	8.47 (7.50)	234 (19.7)	17.24 (8.91)
Missing	32 (2.1)		29 (2.4)	
Gender				
Male	849 (56.9)	9.65 (7.90)	703 (59.2)	17.87 (8.74)
Female	644 (43.1)	9.50 (7.41)	485 (40.8)	17.80 (8.41)
Age group, y				
<65	133 (8.9)	11.96 (8.05)	97 (8.2)	17.44 (8.71)
65‐69	175 (11.7)	9.07 (7.46)	148 (12.5)	16.45 (8.70)
70‐74	253 (16.9)	8.72 (7.90)	212 (17.8)	16.31 (8.73)
75‐79	352 (23.6)	9.34 (7.21)	279 (23.5)	18.07 (8.60)
80+	580 (38.8)	9.73 (7.78)	452 (38.0)	18.96 (8.36)
Informant relationship				
Spouse/partner	1011 (67.7)	9.86 (7.81)	965 (81.2)	17.55 (8.59)
Other	231 (15.5)	10.63 (8.13)	223 (18.8)	19.09 (8.58)
No informant participating	251 (16.8)	7.53 (6.34)		N/A
Mood				
Depressed (GDS‐10 4‐10)	435 (29.1)	12.45 (7.96)	344 (29.0)	19.09 (8.59)
Not depressed (GDS‐10 0‐3)	1017 (68.1)	8.30 (7.15)	815 (68.6)	17.23 (8.55)
Missing	41 (2.7)		29 (2.4)	
Functional ability level				
FAQ 0 level 1	136 (9.1)		31 (2.6)	
FAQ 1‐5 level 2	423 (28.3)		92 (7.7)	
FAQ 6‐9 level 3	287 (19.2)		100 (8.4)	
FAQ 10‐17 level 4	397 (26.6)		329 (27.7)	
FAQ 18‐25 level 5	188 (12.6)		382 (32.2)	
FAQ 26‐33 level 6	62 (4.2)		254 (21.4)	

Abbreviations: FAQ, Functional Activities Questionnaire; FAQ‐I, informant‐rated FAQ; FAQ‐S, self‐rated FAQ; GDS‐10, Geriatric Depression Scale‐10.

### 3.1 Self‐rated functional ability and living well measures

**Figure 1:**
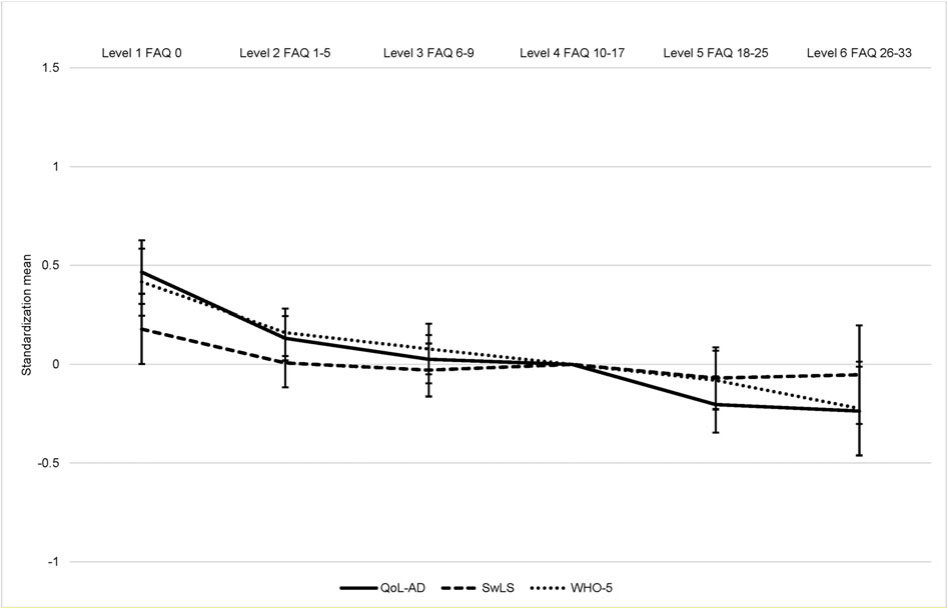
Self‐rated functional ability level by standardised scores on self‐rated living well measures. FAQ, Functional Activities Questionnaire; QoL‐AD, Quality of Life in Alzheimer's Disease; SwLS, Satisfaction with Life Scale; WHO‐5, World Health Organization‐Five Well‐being Index [Colour figure can be viewed at wileyonlinelibrary.com]

Table 2 reports unadjusted and adjusted coefficients between the six FAQ‐S levels and the self‐rated living well measures. Those whose self‐ratings placed them in the most functionally impaired group had lower QoL‐AD, SwLS, and WHO‐5 scores compared with those in the least functionally impaired group. Coefficients reduced after adjusting for sociodemographic and diagnostic factors and slightly increased after further adjustment for ACE‐III score. The largest reduction in coefficients was after adjusting for GDS‐10 score. Figure 1 shows standardised scores for self‐rated living well measures by FAQ‐S level adjusting for sociodemographic and diagnostic factors, ACE‐III, and GDS‐10. For FAQ‐S, coefficients showed a similar declining pattern for QoL‐AD and WHO‐5, indicating that these measures had a similar relationship with functional ability irrespective of FAQ level. SwLS showed the least change across functional ability level with coefficients around zero, though across all FAQ levels, confidence intervals overlapped; see Table S2.

**Table 2:** Relationship of self‐rated functional ability to scores on living well measures: Unadjusted and adjusted unstandardised regression coefficients and 95% confidence intervals

		QoL‐AD	SwLS	WHO‐5
Model 1 Unadjusted	FAQ‐S 0	4.67 (3.56 to 5.78)***	2.76 (1.58 to 3.93)***	13.55 (9.64 to 17.45)***
	FAQ‐S 1‐5	1.84 (1.06 to 2.62)***	1.00 (0.18 to 1.82)*	5.79 (3.04 to 8.53)***
	FAQ‐S 6‐9	0.87 (0.01 to 1.73)	0.53 (−0.38 to 1.44)	3.65 (0.60 to 6.70)*
	FAQ‐S 10‐17	ref	ref	ref
	FAQ‐S 18‐25	−2.36 (−3.35 to −1.38)***	−1.32 (−2.37 to −0.28)*	−3.83 (−7.32 to −0.35)*
	FAQ‐S 26‐33	−2.29 (−3.81 to −0.77)**	−0.94 (−2.55 to 0.67)	−5.10 (−10.47 to 0.28)
Model 2 Adjusted for age, sex, diagnosis, and education	FAQ‐S 0	4.31 (3.21 to 5.41)***	2.30 (1.13 to 3.46)***	12.30 (8.40 to 16.20)***
	FAQ‐S 1‐5	1.50 (0.73 to 2.27)***	0.58 (−0.24 to 1.39)	4.65 (1.91 to 7.39)***
	FAQ‐S 6‐9	0.71 (−0.14 to 1.56)	0.26 (−0.64 to 1.15)	2.93 (−0.09 to 5.95)
	FAQ‐S 10‐17	ref	ref	ref
	FAQ‐S 18‐25	−2.03 (−3.00 to −1.06)***	−1.09 (−2.12 to −0.06)*	−3.17 (−6.61 to 0.28)
	FAQ‐S 26‐33	−1.93 (−3.42 to −0.43)*	−0.63 (−2.21 to 0.95)	−4.40 (−9.71 to 0.92)
Model 3 Adjusted for age, sex, diagnosis, education, and ACE‐III	FAQ‐S 0	4.53 (3.42 to 5.65)***	2.59 (1.42 to 3.76)***	14.20 (10.28 to 18.12)***
	FAQ‐S 1‐5	1.69 (0.90 to 2.47)***	0.82 (−0.00 to 1.65)	6.24 (3.46 to 9.01)***
	FAQ‐S 6‐9	0.80 (−0.05 to 1.66)	0.39 (−0.51 to 1.28)	3.75 (0.74 to 6.75)**
	FAQ‐S 10‐17	ref	ref	ref
	FAQ‐S 18‐25	−2.25 (−3.24 to −1.26)***	−1.38 (−2.42 to −0.33)*	−5.00 (−8.48 to −1.53)**
	FAQ‐S 26‐33	−2.28 (−3.81 to −0.76)**	−1.10 (−2.71 to 0.51)	−7.41 (−12.81 to −2.01)**
Model 4 Adjusted for age, sex, diagnosis, education, ACE‐III, and GDS‐10	FAQ‐S 0	2.79 (1.83 to 3.74)***	1.08 (0.02 to 2.15)*	8.54 (5.07 to 12.01)***
	FAQ‐S 1‐5	0.80 (0.13 to 1.47)*	0.05 (−0.70 to 0.81)	3.36 (0.92 to 5.80)**
	FAQ‐S 6‐9	0.14 (−0.59 to 0.87)	−0.19 (−1.00 to 0.62)	1.59 (−1.05 to 4.23)
	FAQ‐S 10‐17	ref	ref	ref
	FAQ‐S 18‐25	−1.22 (−2.07 to −0.37)**	−0.48 (−1.43 to 0.46)	−1.66 (−4.73 to 1.42)
	FAQ‐S 26‐33	−1.39 (−2.69 to −0.08)*	−0.32 (−1.78 to 1.13)	−4.50 (−9.22 to 0.22)

Abbreviations: ACE‐III, Addenbrooke's Cognitive Examination‐III; FAQ‐S, self‐rated Functional Activities Questionnaire; GDS‐10, Geriatric Depression Scale‐10; QoL‐AD, Quality of Life in Alzheimer's Disease; SwLS, Satisfaction with Life Scale; WHO‐5, World Health Organization‐Five Well‐being Index.

* *P* ≤ .05.

** *P* ≤ .01.

*** *P* ≤ .001.

### 3.2 Informant‐rated functional ability and living well measures

**Figure 2:**
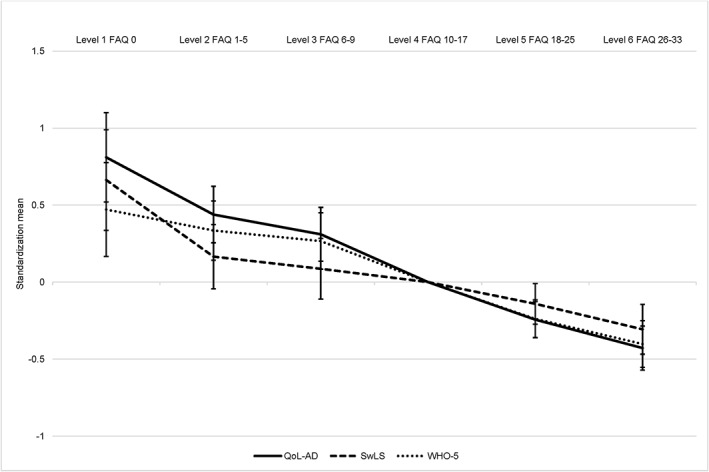
Informant‐rated functional ability level by standardised scores on informant‐rated living well measures. FAQ, Functional Activities Questionnaire; QoL‐AD, Quality of Life in Alzheimer's Disease; SwLS, Satisfaction with Life Scale; WHO‐5, World Health Organization‐Five Well‐being Index

Table 3 reports the unadjusted and adjusted coefficients between the six FAQ‐I levels and the informant‐rated living well measures. Compared with those with the least informant‐rated functional impairment, those in the most functionally impaired group had lower QoL‐AD, SwLS, and WHO‐5 scores. After adjusting for sociodemographic and diagnostic factors, the differences were largely unchanged. Coefficients increased slightly after further adjustment for ACE‐III score, while adjusting for GDS‐10 score produced a slight reduction. Adjusting for RSS score produced the largest reduction in coefficients. Figure 2 shows standardised informant‐rated living well measures by FAQ‐I level adjusting for sociodemographic and diagnostic factors, ACE‐III, GDS‐10, and RSS. For FAQ‐I, QoL‐AD showed the biggest difference between the most and least functionally impaired participants. The direction of coefficients was similar for all three living well measures, although across all FAQ levels, confidence intervals overlapped; see Table S3.

**Table 3:** Relationship of informant‐rated functional ability to scores on living well measures: Unadjusted and adjusted unstandardised regression coefficients and 95% confidence intervals

		QoL‐AD	SwLS	WHO‐5
Model 1 Unadjusted	FAQ‐I 0	6.82 (4.92 to 8.73)***	5.75 (3.30 to 8.20)***	15.56 (8.61 to 22.51)***
	FAQ‐I 1‐5	4.42 (3.23 to 5.62)***	2.76 (1.22 to 4.30)***	12.48 (8.11 to 16.84)***
	FAQ‐I 6‐9	2.62 (1.46 to 3.78)***	1.37 (−0.12 to 2.86)	7.63 (3.41 to 11.86)***
	FAQ‐I 10‐17	ref	ref	ref
	FAQ‐I 18‐25	−2.38 (−3.14 to −1.61)***	−1.66 (−2.64 to −0.68)***	−7.70 (−10.48 to −4.91)***
	FAQ‐I 26‐33	−4.28 (−5.13 to −3.44)***	−3.38 (−4.47 to −2.28)***	−12.98 (−16.07 to −9.88)***
Model 2 Adjusted for age, sex, diagnosis, and education	FAQ‐I 0	6.80 (4.89 to 8.72)***	6.20 (3.79 to 8.60)***	15.41 (8.47 to 22.35)***
	FAQ‐I 1‐5	4.36 (3.16 to 5.56)***	2.66 (1.14 to 4.17)***	12.10 (7.74 to 16.46)***
	FAQ‐I 6‐9	2.60 (1.44 to 3.76)***	1.23 (−0.23 to 2.69)	7.52 (3.31 to 11.73)***
	FAQ‐I 10‐17	ref	ref	ref
	FAQ‐I 18‐25	−2.40 (−3.16 to −1.63)***	−1.75 (−2.72 to −0.78)***	−7.58 (−10.36 to −4.79)***
	FAQ‐I 26‐33	−4.17 (−5.02 to −3.31)***	−3.33 (−4.42 to −2.25)***	−12.25 (−15.36 to −9.15)***
Model 3 Adjusted for age, sex, diagnosis, education, and ACE‐III	FAQ‐I 0	7.03 (5.11 to 8.96)***	6.52 (4.10 to 8.95)***	16.63 (9.66 to 23.60)***
	FAQ‐I 1‐5	4.49 (3.28 to 5.70)***	2.84 (1.31 to 4.36)***	12.78 (8.41 to 17.16)***
	FAQ‐I 6‐9	2.68 (1.51 to 3.84)***	1.34 (−0.12 to 2.80)	7.94 (3.73 to 12.14)***
	FAQ‐I 10‐17	ref	ref	ref
	FAQ‐I 18‐25	−2.51 (−3.29 to −1.74)***	−1.91 (−2.89 to −0.94)***	−8.19 (−11.00 to −5.38)***
	FAQ‐I 26‐33	−4.51 (−5.43 to −3.59)***	−3.83 (−5.00 to −2.66)***	−14.10 (−17.44 to −10.75)***
Model 4 Adjusted for age, sex, diagnosis, education, ACE‐III, and GDS‐10	FAQ‐I 0	6.93 (5.09 to 8.77)***	6.41 (4.07 to 8.74)***	16.23 (9.65 to 22.81)***
	FAQ‐I 1‐5	4.34 (3.18 to 5.49)***	2.65 (1.19 to 4.12)***	12.14 (8.02 to 16.27)***
	FAQ‐I 6‐9	2.56 (1.45 to 3.67)***	1.20 (−0.20 to 2.61)	7.46 (3.49 to 11.42)***
	FAQ‐I 10‐17	ref	ref	ref
	FAQ‐I 18‐25	−2.44 (−3.18 to −1.70)***	−1.83 (−2.77 to −0.89)***	−7.90 (−10.56 to −5.25)***
	FAQ‐I 26‐33	−4.30 (−5.18 to −3.42)***	−3.58 (−4.71 to −2.45)***	−13.24 (−16.40 to −10.08)***
Model 5 Adjusted for age, sex, diagnosis, education, ACE‐III, GDS‐10, and RSS	FAQ‐I 0	4.69 (2.99 to 6.40)***	4.54 (2.25 to 6.82)***	9.60 (3.30 to 15.90)**
	FAQ‐I 1‐5	2.57 (1.48 to 3.65)***	1.18 (−0.27 to 2.63)	6.91 (2.93 to 10.88)***
	FAQ‐I 6‐9	1.78 (0.76 to 2.80)***	0.55 (−0.82 to 1.92)	5.14 (1.39 to 8.89)**
	FAQ‐I 10‐17	ref	ref	ref
	FAQ‐I 18‐25	−1.46 (−2.14 to −0.77)***	−1.01 (−1.93 to −0.09)*	−4.99 (−7.53 to −2.45)***
	FAQ‐I 26‐33	−2.50 (−3.34 to −1.66)***	−2.08 (−3.21 to −0.95)***	−7.91 (−11.01 to −4.81)***

Abbreviations: ACE‐III, Addenbrooke's Cognitive Examination‐III; FAQ‐I, informant‐rated Functional Activities Questionnaire; GDS‐10, Geriatric Depression Scale‐10; QoL‐AD, Quality of Life in Alzheimer's Disease; RSS, Relatives' Stress Scale; SwLS, Satisfaction with Life Scale; WHO‐5, World Health Organization‐Five Well‐being Index.

* *P* ≤ .05.

** *P* ≤ .01.

*** *P* ≤ .001.

### 3.3 Impact of missing data on the results

The percentage of missing data was between 1.3% and 10.5% across all domains for people with dementia and between 0.2% and 6.8% for informants (see Table S1). Coefficients were generally similar to the complete case analysis, but standard errors reduced after multiple imputations. Imputation did not alter the relationships but improved the precision of estimates.

## 4 DISCUSSION

This large cohort study of community‐dwelling people with dementia and their informants across Great Britain investigated the association between perceived functional ability and three measures serving as indices of living well, the QoL‐AD scale, SwLS, and WHO‐5. This study extends previous research, which has primarily focussed only on the association between functional ability and QoL.^25^, ^40^ The results show a decreasing pattern in living well scores, particularly for QoL and well‐being, from the least to most functionally impaired participants; there was a gradual decline with no clear transition point, and confidence intervals overlapped between the levels. This pattern remained for self‐ratings and informant ratings after correcting for cognition and for sociodemographic and diagnostic factors. Importantly, even subtle perceived functional impairments influenced perceptions of living well. The findings indicate that for self‐ratings and informant ratings, people with dementia with the least functional impairments had greater capability to live well than those with the most functional impairment.

Increased depression in people with dementia attenuated the relationship between self‐rated functional ability and living well measures. Those with higher depression scores reported more impaired functional ability by a difference of four points on self‐rated and two points on informant‐rated functional ability. This may arise because of the potent influence of depression on functional ability and living well. The finding corroborates earlier studies where depression was found to separately influence functional ability and self‐rated indicators of living well.^31^, ^33^, ^35^, ^36^, ^39^, ^40^, ^45^, ^59^ The majority of previous studies, however, have employed diagnostic classifications or informant ratings of depression. For example, a recent study found that people with dementia who had a formal diagnosis of depression rated themselves as *less* impaired in functional ability than people with dementia who did not have a diagnosis of depression.^28^ However, they did not report current levels of depression, so it is possible that ameliorative treatment may have influenced the results. There has been relatively little research investigating the relationship between self‐rated depression and functional ability in people with dementia, primarily because of a scarcity of studies employing self‐rated measures of functional ability.^2^ Previously, and consistent with the current findings, we found a significant association between self‐rated depression and self‐rated functional ability,^60^ where those who were more depressed rated their functional ability as more impaired. It may be that people who are depressed are likely to overestimate functional impairments because of negative thought processes leading to an overall negative view of self, though in contrast “depressive realism” may lead to a more realistic appraisal.^61^ As either interpretation could explain our current findings, further research is needed to clarify how self‐rated depression interacts with self‐rated and objective functional ability in people with dementia to fully delineate the relationship.

Greater carer stress attenuated the associations between informant‐rated functional ability and living well measures. Consistent with previous studies, informants experiencing higher perceived stress tended to rate the person with dementia as having poorer ability to live well and more functional impairment.^40^, ^45^, ^46^, ^62^, ^63^, ^64^, ^65^ Interestingly, informants had relatively mild levels of perceived stress, suggesting that even mild carer stress may influence informant ratings of functional ability and living well. This raises questions about the reliability and validity of relying solely on informants to provide judgements of functional ability, particularly as most carers report some level of stress due to their caring responsibilities,^66^, ^67^ and informant ratings are generally the only source of information collected in functional evaluations during clinical and research assessments.^2^, ^45^ Levels of carer stress need to be considered in future research and clinical assessments that employ informant ratings. Our findings indicate that it is necessary to account for the influence of carer stress on judgements of functional ability and living well.

Standardising the scores on living well measures allowed for direct comparison of the impact of functional ability on each measure. While there were differences between the living well measures at each level of informant‐rated functional ability, overall, there was a consistent decline in effect sizes between those with the most and least perceived functional ability. For self‐rated living well, QoL and well‐being declined consistently, while satisfaction with life was generally stable across the functional ability levels, which fits with the suggestion that satisfaction with life may be relatively stable; for example, there is little quantitative difference between the scores of university students and healthy older people.^68^ The decreasing pattern in living well scores from the least to most functionally impaired participants supports disablement process theory where increasing disability leads to reduced QoL^12^ and is also consistent with the emphasis on depression and functional ability in Lawton's dementia‐specific model of QoL.^69^ The current study extends this to well‐being, although after controlling for depression, self‐rated satisfaction with life was not affected by changes in functional ability.

The study had some limitations. Despite functional difficulties being required for a diagnosis of dementia, ratings by 136 people with dementia and 31 informants reported no functional impairment. This was unrelated to age or type of dementia as most of those with “no functional impairment” were over 80 and had Alzheimer's disease. The FAQ has been described as one of the more sensitive functional questionnaires for people with early‐stage dementia^44^, ^70^ with studies recommending its use to distinguish mild cognitive decline from dementia.^71^, ^72^ However, while the brevity of the FAQ ensures suitability for people with dementia, it is likely that important aspects of functional ability have been omitted from the measure, such as those included in the Amsterdam Instrumental Activity of Daily Living Questionnaire;^73^, ^74^ we previously modified the FAQ to include a question on telephone use.^30^ Our findings suggest that for some people with early‐stage dementia, the FAQ may not have adequate sensitivity to identify perceived functional difficulties. The QoL‐AD was used to assess QoL. This measure was designed for people with Alzheimer's disease and may not be sensitive to measure QoL in other dementias, though our meta‐analysis found it is used extensively across all dementias.^40^ While IDEAL is a large study of people with dementia,^25^ the cohort consisted almost exclusively of white British participants, which limits the ability to extrapolate to other cultural or ethnic groups. We used only cross‐sectional data from the IDEAL study; this does not address the relationship of functional ability and living well measures over time or allow prediction of long‐term change in both functional ability and living well. These questions will be addressed once longitudinal data are available.

## 5 CONCLUSION

We found evidence that people with dementia who have the least perceived functional impairments report better QoL, satisfaction with life, and well‐being than those with the greatest perceived functional impairment. Perceived difficulties in instrumental ADL had a detrimental effect on the ability of people with dementia to live well even at low levels of functional impairment. The relationship between functional ability and living well was generally linear, indicating that as difficulties with everyday activities increase, the ability to live well decreases. Importantly, the study found that standardising QoL, satisfaction with life, and well‐being scores resulted in effect sizes for each that were equivalent between the three measures. Therefore, functional ability contributes to the ability of people with dementia to live well. Depression in people with dementia and carer stress in informants were confirmed as confounding factors that negatively influenced ratings of functional ability and measures of living well. Depression in people with dementia and carer stress in informants need to be routinely considered in future research studies and clinical assessments to help facilitate accurate judgements of functional ability and living well. Longitudinal studies are needed to elucidate whether the association between functional ability and living well remains as dementia severity increases further.

## ETHICS STATEMENT

The authors assert that all procedures contributing to this work comply with the ethical standards of the relevant national and institutional committees on human experimentation and with the Helsinki Declaration of 1975, as revised in 2008.

## CONFLICT OF INTEREST

None declared.

## AUTHOR CONTRIBUTIONS

All authors were involved in the original conception and design of the IDEAL study. A.M. is responsible for the data analysis and interpretation and drafting the article. S.M.N., C.Q., J.M.R., R.G.M., and L.C. contributed to the critical revision of the article and approved the version to be published.

## DATA AVAILABILITY STATEMENT

The IDEAL data will be deposited with the UK Data Archive upon completion of the study in March 2020. Details on how the data can be accessed after this date will be made available on the project website www.idealproject.org.uk.

## Supporting information


**Table S1**. Demographic information and mean scores on all measures
**Table S2**. Relationship of self‐rated functional ability to scores on living well measures: unadjusted and adjusted standardised regression coefficients and 95% confidence intervals
**Table S3**. Relationship of informant‐rated functional ability to scores on living well measures: unadjusted and adjusted standardised regression coefficients and 95% confidence intervals

## References

[1] Agüero‐Torres H , Fratiglioni L , Guo Z , Viitanen M , von Strauss E , Winblad B . Dementia is the major cause of functional dependence in the elderly: 3‐year follow‐up data from a population‐based study. Am J Public Health. 1998;88(10):1452‐1456. 9772843 10.2105/ajph.88.10.1452 PMC1508485

[2] Martyr A , Clare L . Executive function and activities of daily living in Alzheimer's disease: a correlational meta‐analysis. Dement Geriatr Cogn Disord. 2012;33(2‐3):189‐203. 22572810 10.1159/000338233

[3] American Psychiatric Association . Diagnostic and Statistical Manual of Mental Disorders. Arlington, VA: American Psychiatric Publishing; 2013.

[4] World Health Organization . International Statistical Classification of Diseases and Related Health Problems, 10th revision (ICD‐10). Geneva, Switzerland: World Health Organ; 1992.

[5] Spector WD , Katz S , Murphy JB , Fulton JP . The hierarchical relationship between activities of daily living and instrumental activities of daily living. J Chronic Dis. 1987;40(6):481‐489. 3597653 10.1016/0021-9681(87)90004-x

[6] Boyle PA , Cohen RA , Paul R , Moser D , Gordon N . Cognitive and motor impairments predict functional declines in patients with vascular dementia. Int J Geriatr Psychiatry. 2002;17(2):164‐169. 11813280 10.1002/gps.539

[7] Green CR , Mohs RC , Schmeidler J , Aryan M , Davis KL . Functional decline in Alzheimer's disease: a longitudinal study. J Am Geriatr Soc. 1993;41(6):654‐661. 8505464 10.1111/j.1532-5415.1993.tb06740.x

[8] Pérès K , Helmer C , Amieva H , et al. Natural history of decline in instrumental activities of daily living performance over the 10 years preceding the clinical diagnosis of dementia: a prospective population‐based study. J Am Geriatr Soc. 2008;56(1):37‐44. 18028344 10.1111/j.1532-5415.2007.01499.x

[9] Njegovan V , Hing MM , Mitchell SL , Molnar FJ . The hierarchy of functional loss associated with cognitive decline in older persons. J Gerontol A Biol Sci Med Sci. 2001;56(10):M638‐M643. 11584037 10.1093/gerona/56.10.m638

[10] Royall DR , Lauterbach EC , Kaufer D , et al. The cognitive correlates of functional status: a review from the Committee on Research of the American Neuropsychiatric Association. J Neuropsychiatry Clin Neurosci. 2007;19(3):249‐265. 17827410 10.1176/jnp.2007.19.3.249

[11] McLaughlin T , Feldman H , Fillit H , et al. Dependence as a unifying construct in defining Alzheimer's disease severity. Alzheimers Dement. 2010;6(6):482‐493. 21044778 10.1016/j.jalz.2009.09.004 PMC3884683

[12] Verbrugge LM , Jette AM . The disablement process. Soc Sci Med. 1994;38(1):1‐14. 8146699 10.1016/0277-9536(94)90294-1

[13] Andersen CK , Wittrup‐Jensen KU , Lolk A , Andersen K , Kragh‐Sørensen P . Ability to perform activities of daily living is the main factor affecting quality of life in patients with dementia. Health Qual Life Outcomes. 2004;2(1):52. 15383148 10.1186/1477-7525-2-52 PMC521495

[14] Logsdon RG , McCurry SM , Teri L . Evidence‐based interventions to improve quality of life for individuals with dementia. Alzheimers Care Today. 2007;8(4):309‐318. 19030120 PMC2585781

[15] O'Rourke HM , Duggleby W , Fraser KD , Jerke L . Factors that affect quality of life from the perspective of people with dementia: a metasynthesis. J Am Geriatr Soc. 2015;63(1):24‐38. 25597556 10.1111/jgs.13178

[16] Pickett J , Bird C , Ballard C , et al. A roadmap to advance dementia research in prevention, diagnosis, intervention, and care by 2025. Int J Geriatr Psychiatry. 2018;33(7):900‐906. 29468724 10.1002/gps.4868 PMC6033035

[17] Department of Health . Living Well With Dementia: A National Dementia Strategy. London: Department of Health; 2009.

[18] US Department of Heath and Human Services . National Plan to Address Alzheimer's Disease. Washington, D.C.: US Department of Heath and Human Services; 2012.

[19] Poulos CJ , Bayer A , Beaupre L , et al. A comprehensive approach to reablement in dementia. Alzheimers Dement (N Y). 2017;3(3):450‐458. 29067351 10.1016/j.trci.2017.06.005 PMC5654482

[20] Institute Of Medicine . Living Well With Chronic Illness: A Call for Public Health Action. Washington: National Academies Press; 2012.

[21] Lorig K , Holman HR , Sobel D , et al. Living a Healthy Life With Chronic Condition: Self‐Management of Heart Disease, Arthritis, Diabetes, Asthma, Bronchitis, Enphysema and Others. Boulder, CO: Bull Publishing Company; 2006.

[22] Clare L , Wu Y‐T , Jones IR , et al. A comprehensive model of factors associated with subjective perceptions of “living well” with dementia: findings from the IDEAL study. Alzheimer Dis Assoc Disord. 2019;33(1):36‐41. 30802227 10.1097/WAD.0000000000000286 PMC6416010

[23] Dodge R , Daly AP , Huyton J , Sanders LD . The challenge of defining wellbeing. Int J Wellbeing. 2012;2(3):222‐235.

[24] Diener E , Chan MY . Happy people live longer: subjective well‐being contributes to health and longevity. Appl Psychol Health Well‐Being. 2011;3(1):1‐43.

[25] Clare L , Nelis SM , Quinn C , et al. Improving the experience of dementia and enhancing active life—living well with dementia: study protocol for the IDEAL study. Health Qual Life Outcomes. 2014;12(1):164. 25433373 10.1186/s12955-014-0164-6 PMC4260182

[26] Silarova B , Nelis SM , Ashworth RM , et al. Protocol for the IDEAL‐2 longitudinal study: following the experiences of people with dementia and their primary carers to understand what contributes to living well with dementia and enhances active life. BMC Public Health. 2018;18(1):1214. 30376832 10.1186/s12889-018-6129-7 PMC6208177

[27] Fitz AG , Teri L . Depression, cognition, and functional ability in patients with Alzheimer's disease. J Am Geriatr Soc. 1994;42(2):186‐191. 8126334 10.1111/j.1532-5415.1994.tb04950.x

[28] Mograbi DC , Morris RG , Fichman HC , et al. The impact of dementia, depression and awareness on activities of daily living in a sample from a middle‐income country. Int J Geriatr Psychiatry. 2018;33(6):807‐813. 28786127 10.1002/gps.4765

[29] Hargrave R , Reed B , Mungas D . Depressive syndromes and functional disability in dementia. J Geriatr Psychiatry Neurol. 2000;13(2):72‐77. 10912728 10.1177/089198870001300205

[30] Martyr A , Clare L , Nelis SM , et al. Verbal fluency and awareness of functional deficits in early‐stage dementia. Clin Neuropsychol. 2012;26(3):501‐519. 22394254 10.1080/13854046.2012.665482

[31] Pearson JL , Teri L , Reifler BV , Raskind MA . Functional status and cognitive impairment in Alzheimer's patients with and without depression. J Am Geriatr Soc. 1989;37(12):1117‐1121. 2592718 10.1111/j.1532-5415.1989.tb06674.x

[32] Martyr A , Clare L . Awareness of functional ability in people with early‐stage dementia. Int J Geriatr Psychiatry. 2018;33(1):31‐38. 28071830 10.1002/gps.4664

[33] Bosboom PR , Alfonso H , Eaton J , Almeida OP . Quality of life in Alzheimer's disease: different factors associated with complementary ratings by patients and family carers. Int Psychogeriatr. 2012;24(5):708‐721. 22244307 10.1017/S1041610211002493

[34] Dourado MC , Sousa MF , Santos RL , et al. Quality of life in mild dementia: patterns of change in self and caregiver ratings over time. Rev Bras Psiquiatr. 2016;38(4):294‐300. 26785107 10.1590/1516-4446-2014-1642 PMC7111349

[35] Gómez‐Gallego M , Gómez‐Amor J , Gómez‐García J . Determinants of quality of life in Alzheimer's disease: perspective of patients, informal caregivers, and professional caregivers. Int Psychogeriatr. 2012;24(11):1805‐1815. 22697366 10.1017/S1041610212001081

[36] Heggie M , Morgan D , Crossley M , et al. Quality of life in early dementia: comparison of rural patient and caregiver ratings at baseline and one year. Dementia. 2012;11(4):521‐541. 24976791 10.1177/1471301211421085 PMC4071058

[37] Ready RE , Ott BR , Grace J . Patient versus informant perspectives of quality of life in mild cognitive impairment and Alzheimer's disease. Int J Geriatr Psychiatry. 2004;19(3):256‐265. 15027041 10.1002/gps.1075

[38] Sheehan BD , Lall R , Stinton C , et al. Patient and proxy measurement of quality of life among general hospital in‐patients with dementia. Aging Ment Health. 2012;16(5):603‐607. 22360734 10.1080/13607863.2011.653955

[39] Woods RT , Nelis SM , Martyr A , et al. What contributes to a good quality of life in early dementia? Awareness and the QoL‐AD: a cross‐sectional study. Health Qual Life Outcomes. 2014;12(1):94. 24919416 10.1186/1477-7525-12-94 PMC4061777

[40] Martyr A , Nelis SM , Quinn C , et al. Living well with dementia: a systematic review and correlational meta‐analysis of factors associated with quality of life, well‐being and life satisfaction in people with dementia. Psychol Med. 2018;48(13):2130‐2139. 29734962 10.1017/S0033291718000405

[41] Sikkes SA , de Lange‐de Klerk ES , Pijnenburg YA , Scheltens P , Uitdehaag BM . A systematic review of Instrumental Activities of Daily Living scales in dementia: room for improvement. J Neurol Neurosurg Psychiatry. 2009;80(1):7‐12. 19091706 10.1136/jnnp.2008.155838

[42] Smyth KA , Neundorfer MM , Koss E , Geldmacher DS , Ogrocki PK , Whitehouse PJ . Quality of life and deficit identification in dementia. Dementia. 2002;1(3):345‐358.

[43] Vasterling JJ , Seltzer B , Foss MW , Vanderbrook V . Unawareness of deficit in Alzheimer's disease: domain‐specific differences and disease correlates. Cogn Behav Neurol. 1995;8(1):26‐32.

[44] Costa PT Jr , Williams TF , Albert MS , et al. Early Identification of Alzheimer's Disease and Related Dementias. Clinical Practice Guideline, Quick Reference for Clinicians, No. 19. Rockville, MD: U.S. Department of Health and Human Services; 1996.

[45] Martyr A , Nelis SM , Clare L . Predictors of perceived functional ability in early‐stage dementia: self‐ratings, informant ratings and discrepancy scores. Int J Geriatr Psychiatry. 2014;29(8):852‐862. 24753076 10.1002/gps.4071

[46] Razani J , Kakos B , Orieta‐Barbalace C , et al. Predicting caregiver burden from daily functional abilities of patients with mild dementia. J Am Geriatr Soc. 2007;55(9):1415‐1420. 17767684 10.1111/j.1532-5415.2007.01307.x PMC2288619

[47] Pfeffer RI , Kurosaki TT , Harrah CH Jr , Chance JM , Filos S . Measurement of functional activities in older adults in the community. J Gerontol. 1982;37(3):323‐329. 7069156 10.1093/geronj/37.3.323

[48] Folstein MF , Folstein SE , McHugh PR . “Mini‐Mental State”. A practical method for grading the cognitive state of patients for the clinician. J Psychiatr Res. 1975;12(3):189‐198. 1202204 10.1016/0022-3956(75)90026-6

[49] Quinn C , Nelis SM , Martyr A , Victor C , Morris RG , Clare L . Influence of positive and negative dimensions of dementia caregiving on caregiver well‐being and satisfaction with life: findings from the IDEAL study. Am J Geriatr Psychiatry. 2019 10.1016/j.jagp.2019.02.005 30917903

[50] Alzheimer's Association . Tools for Early Identification, Assessment and Treatment for People With Alzheimer's Disease and Dementia. Bloomington, MN: National Chronic Care Consortium and the Alzheimer's Association; 2003.

[51] Logsdon RG , Gibbons LE , McCurry SM , Teri L . Quality of life in Alzheimer's disease: patient and caregiver reports In: Albert SM, Logsdon RG, eds. Assessing Quality of Life in Dementia. New York: Springer; 2000:17‐30.

[52] Diener E , Emmons RA , Larsen RJ , Griffin S . The Satisfaction With Life Scale. J Pers Assess. 1985;49(1):71‐75. 16367493 10.1207/s15327752jpa4901_13

[53] Bech P . Measuring the dimension of psychological general well‐being by the WHO‐5. Qual Life Newslett. 2004;15‐16.

[54] Topp CW , Ostergaard SD , Sondergaard S , Bech P . The WHO‐5 Well‐Being Index: a systematic review of the literature. Psychother Psychosom. 2015;84(3):167‐176. 25831962 10.1159/000376585

[55] Hsieh S , Schubert S , Hoon C , Mioshi E , Hodges JR . Validation of the Addenbrooke's Cognitive Examination III in frontotemporal dementia and Alzheimer's disease. Dement Geriatr Cogn Disord. 2013;36(3‐4):242‐250. 23949210 10.1159/000351671

[56] Almeida OP , Almeida SA . Short versions of the Geriatric Depression Scale: a study of their validity for the diagnosis of a major depressive episode according to ICD‐10 and DSM‐IV. Int J Geriatr Psychiatry. 1999;14(10):858‐865. 10521885 10.1002/(sici)1099-1166(199910)14:10<858::aid-gps35>3.0.co;2-8

[57] Greene JG , Smith R , Gardiner M , Timbury GC . Measuring behavioural disturbance of elderly demented patients in the community and its effects on relatives: a factor analytic study. Age Ageing. 1982;11(2):121‐126. 7102472 10.1093/ageing/11.2.121

[58] Rubin DB . Multiple imputation after 18+ years. J Am Stat Assoc. 1996;91(434):473‐489.

[59] Sands LP , Ferreira P , Stewart AL , Brod M , Yaffe K . What explains differences between dementia patients' and their caregivers' ratings of patients' quality of life? Am J Geriatr Psychiatry. 2004;12(3):272‐280. 15126228

[60] Clare L , Nelis SM , Martyr A , et al. The influence of psychological, social and contextual factors on the expression and measurement of awareness in early‐stage dementia: testing a biopsychosocial model. Int J Geriatr Psychiatry. 2012;27(2):167‐177. 21425345 10.1002/gps.2705

[61] Alloy LB , Abramson LY . Judgment of contingency in depressed and nondepressed students: sadder but wiser? J Exp Psychol Gen. 1979;108(4):441‐485. 528910 10.1037//0096-3445.108.4.441

[62] Andrieu S , Coley N , Rolland Y , et al. Assessing Alzheimer's disease patients' quality of life: discrepancies between patient and caregiver perspectives. Alzheimers Dement. 2016;12(4):427‐437. 26602086 10.1016/j.jalz.2015.09.003

[63] Black BS , Johnston D , Morrison A , Rabins PV , Lyketsos CG , Samus QM . Quality of life of community‐residing persons with dementia based on self‐rated and caregiver‐rated measures. Qual Life Res. 2012;21(8):1379‐1389. 22038392 10.1007/s11136-011-0044-z PMC3296880

[64] Conde‐Sala JL , Garre‐Olmo J , Turró‐Garriga O , López‐Pousa S , Vilalta‐Franch J . Factors related to perceived quality of life in patients with Alzheimer's disease: the patient's perception compared with that of caregivers. Int J Geriatr Psychiatry. 2009;24(6):585‐594. 19031477 10.1002/gps.2161

[65] Naglie G , Hogan DB , Krahn M , et al. Predictors of family caregiver ratings of patient quality of life in Alzheimer disease: cross‐sectional results from the Canadian Alzheimer's Disease Quality of Life Study. Am J Geriatr Psychiatry. 2011;19(10):891‐901. 21946805 10.1097/JGP.0b013e3182006a7f PMC3267778

[66] Brodaty H , Donkin M . Family caregivers of people with dementia. Dialogues Clin Neurosci. 2009;11(2):217‐228. 19585957 10.31887/DCNS.2009.11.2/hbrodaty PMC3181916

[67] Donaldson C , Tarrier N , Burns A . The impact of the symptoms of dementia on caregivers. Br J Psychiatry. 1997;170(1):62‐68. 9068778 10.1192/bjp.170.1.62

[68] Pavot W , Diener E . Review of the satisfaction with life scale. Psychol Assess. 1993;5(2):164‐172.

[69] Lawton MP . Quality of life in Alzheimer disease. Alzheimer Dis Assoc Disord. 1994;8(Suppl 3):138‐150. 7999340

[70] Karagiozis H , Gray S , Sacco J , Shapiro M , Kawas C . The Direct Assessment of Functional Abilities (DAFA): a comparison to an indirect measure of instrumental activities of daily living. Gerontologist. 1998;38(1):113‐121. 9499659 10.1093/geront/38.1.113

[71] Brown PJ , Devanand DP , Liu X , Caccappolo E . Functional impairment in elderly patients with mild cognitive impairment and mild Alzheimer disease. Arch Gen Psychiatry. 2011;68(6):617‐626. 21646578 10.1001/archgenpsychiatry.2011.57 PMC3682408

[72] Teng E , Becker BW , Woo E , Knopman DS , Cummings JL , Lu PH . Utility of the functional activities questionnaire for distinguishing mild cognitive impairment from very mild Alzheimer disease. Alzheimer Dis Assoc Disord. 2010;24(4):348‐353. 20592580 10.1097/WAD.0b013e3181e2fc84 PMC2997338

[73] Jutten RJ , Peeters CFW , Leijdesdorff SMJ , et al. Detecting functional decline from normal aging to dementia: development and validation of a short version of the Amsterdam IADL Questionnaire. Alzheimers Dement (Amst). 2017;8(1):26‐35. 28462387 10.1016/j.dadm.2017.03.002 PMC5403784

[74] Sikkes SA , de Lange‐de Klerk ES , Pijnenburg YA , Uitdehaag BM , Scheltens P . A new informant‐based questionnaire for instrumental activities of daily living in dementia. Alzheimers Dement. 2012;8(6):536‐543. 23102123 10.1016/j.jalz.2011.08.006

