# Supplementary material for: The relationship between perceived functional difficulties and the ability to live well with mild‐to‐moderate dementia: Findings from the IDEAL programme
Source: Int J Geriatr Psychiatry. 2019 May 20;34(8):1251–61. doi: 10.1002/gps.5128 (PMC6767698; doi:10.1002/gps.5128)
Supplement: Supplementary file 1 — Table S1. Demographic information and mean scores on all measures Table S2. Relationship of self‐rated functional ability to scores on living well measures: unadjusted and adjusted standardised regression coefficients and 95% confidence intervals Table S3. Relationship of informant‐rated functional ability to scores on living well measures: unadjusted and adjusted standardised regression coefficients and 95% confidence intervals [file GPS-34-1251-s001.docx]

Supplementary Table 1. Demographic information and mean scores on all measures

|  | Person with dementia | |  | Informant | |
| --- | --- | --- | --- | --- | --- |
|  | N=1493 | |  | N=1188 | |
|  | n (%) | Missing |  | n (%) | Missing |
| Female | 646 (43.7) | 0 | Female | 816 (68.7) | 0 |
|  |  |  | Spouse/partner | 965 (81.2) | 0 |
|  |  |  | Other | 223 (18.8) | 0 |
|  |  |  |  |  |  |
|  | Mean (SD) | Missing |  | Mean (SD) | Missing |
| Age | 76.30 (8.51) | 0 | Age | 69.11 (11.06) | 2 (0.2%) |
| QoL-AD | 36.79 (5.93) | 129 (8.6%) | QoL-AD | 33.67 (5.87) | 81 (6.8%) |
| SwLS | 26.07 (6.10) | 31 (2.1%) | SwLS | 20.86 (6.96) | 21 (1.8%) |
| WHO-5 | 60.99 (20.58) | 19 (1.3%) | WHO-5 | 49.80 (20.53) | 19 (1.6%) |
| FAQ | 9.59 (7.69) | 0 | FAQ | 17.84 (8.61) | 0 |
| ACE-III | 69.35 (13.13) | 94 (6.3%) | RSS | 19.17 (9.84) | 56 (4.7%) |
| GDS-10 | 2.65 (2.28) | 156 (10.5%) |  |  |  |

Note: Addenbrooke’s Cognitive Examination-III, ACE-III; Functional Activities Questionnaire, FAQ; Geriatric Depression Scale-10, GDS-10; Quality of Life in Alzheimer’s Disease, QoL-AD; Relatives’ Stress Scale, RSS; Satisfaction with Life Scale, SwLS; WHO-5, World Health Organization-Five Well-being Index.

Supplementary Table 2. Relationship of self-rated functional ability to scores on living well measures: unadjusted and adjusted standardised regression coefficients and 95% confidence intervals

|  |  | QoL-AD | SwLS | WHO-5 |
| --- | --- | --- | --- | --- |
| Model 1  Unadjusted | FAQ-S 0 | .78 (.60, .97)*** | .46 (.26, .65)*** | .66 (.47, .85)*** |
|  | FAQ-S 1-5 | .31 (.18, .44)*** | .17 (.03, .30)* | .28 (.15, .42)*** |
|  | FAQ-S 6-9 | .15 (-.00, .29) | .09 (-.06, .24) | .18 (.03, .33)* |
|  | FAQ-S 10-17 | ref | ref | ref |
|  | FAQ-S 18-25 | -.40 (-.56, -.23)*** | -.22 (-.39, -.05)* | -.19 (-.36, -.02)* |
|  | FAQ-S 26-33 | -.38 (-.64, -.13)** | -.15 (-.42, .11) | -.25 (-.51, .01) |
| Model 2  Adjusted for age, sex, diagnosis and education | FAQ-S 0 | .72 (.54, .91)*** | .38 (.19, .57)*** | .60 (.41, .77)*** |
|  | FAQ-S 1-5 | .25 (.12, .38)*** | .10 (-.04, .23) | .23 (.09, .36)*** |
|  | FAQ-S 6-9 | .12 (-.03, .26) | .04 (-.11, .19) | .14 (-.00, .29) |
|  | FAQ-S 10-17 | ref | ref | ref |
|  | FAQ-S 18-25 | -.34 (-.51, -.18)*** | -.18 (-.35, -.01)* | -.15 (-.32, .01) |
|  | FAQ-S 26-33 | -.32 (-.57, -.07)* | -.10 (-.37, .16) | -.21 (-.47, .05) |
| Model 3  Adjusted for age, sex, diagnosis, education, and ACE-III | FAQ-S 0 | .76 (.57, .95)*** | .43 (.23, .62)*** | .69 (.50, .88)*** |
|  | FAQ-S 1-5 | .28 (.15, .42)*** | .14 (-.00, .27) | .30 (.17, .44)*** |
|  | FAQ-S 6-9 | .13 (-.01, .28) | .06 (-.09, .21) | .18 (.04, .33)* |
|  | FAQ-S 10-17 | ref | ref | ref |
|  | FAQ-S 18-25 | -.38 (-.55, -.21)*** | -.23 (-.40, -.06)* | -.24 (-.41, -.07)** |
|  | FAQ-S 26-33 | -.38 (-.64, -.12)** | -.18 (-.45, .08) | -.36 (-.62, -.10)** |
| Model 4  Adjusted for age, sex, diagnosis, education, ACE-III and GDS-10 | FAQ-S 0 | .47 (.31, .63)*** | .18 (.00, .36)* | .42 (.25, .58)*** |
|  | FAQ-S 1-5 | .13 (.02, .25)* | .01 (-.12, .13) | .16 (.05, .28)** |
|  | FAQ-S 6-9 | .02 (-.10, .15) | -.03 (-.17, .10) | .08 (-.05, .21) |
|  | FAQ-S 10-17 | ref | ref | ref |
|  | FAQ-S 18-25 | -.21 (-.35, -.06)** | -.08 (-.24, .08) | -.08 (-.23, .07) |
|  | FAQ-S 26-33 | -.23 (-.45, -.01)* | -.05 (-.29, .19) | -.22 (-.45, .01) |

Note: Addenbrooke’s Cognitive Examination-III, ACE-III; Functional Activities Questionnaire, FAQ; Geriatric Depression Scale-10, GDS-10; Quality of Life in Alzheimer’s Disease, QoL-AD; Satisfaction with Life Scale, SwLS; WHO-5, World Health Organization-Five Well-being Index. * *p*≤.05, ** *p*≤.01, *** *p*≤.001

Supplementary Table 3. Relationship of informant-rated functional ability to scores on living well measures: unadjusted and adjusted standardised regression coefficients and 95% confidence intervals

|  |  | QoL-AD | SwLS | WHO-5 |
| --- | --- | --- | --- | --- |
| Model 1  Unadjusted | FAQ-I 0 | 1.16 (.84, 1.49)*** | .83 (.47, 1.18)*** | .76 (.42, 1.10)*** |
|  | FAQ-I 1-5 | .75 (.55, .96)*** | .40 (.18, .62)*** | .61 (.40, .82)*** |
|  | FAQ-I 6-9 | .45 (.25, .64)*** | .20 (-.02, .41) | .37 (.17, .58)*** |
|  | FAQ-I 10-17 | ref | ref | ref |
|  | FAQ-I 18-25 | -.40 (-.53, -.27)*** | -.24 (-.38, -.10)*** | -.37 (-.51, -.24)*** |
|  | FAQ-I 26-33 | -.72 (-.87, -.58)*** | -.49 (-.64, -.33)*** | -.63 (-.78, -.48)*** |
| Model 2  Adjusted for age, sex, diagnosis and education | FAQ-I 0 | 1.16 (.83, 1.48)*** | .89 (.54, 1.24)*** | .75 (.41, 1.09)*** |
|  | FAQ-I 1-5 | .74 (.54, .95)*** | .38 (.16, .60)*** | .59 (.38, .80)*** |
|  | FAQ-I 6-9 | .44 (.25, .64)*** | .18 (-.03, .39) | .37 (.16, .57)*** |
|  | FAQ-I 10-17 | ref | ref | ref |
|  | FAQ-I 18-25 | -.41 (-.54, -.28)*** | -.25 (-.39, -.11)*** | -.37 (-.50, -.23)*** |
|  | FAQ-I 26-33 | -.70 (-.85, -.56)*** | -.48 (-.63, -.32)*** | -.60 (-.75, -.45)*** |
| Model 3  Adjusted for age, sex, diagnosis, education, and ACE-III | FAQ-I 0 | 1.20 (.87, 1.53)*** | .94 (.59, 1.29)*** | .81 (.47, 1.15)*** |
|  | FAQ-I 1-5 | .77 (.56, .97)*** | .41 (.19, .63)*** | .62 (.41, .84)*** |
|  | FAQ-I 6-9 | .46 (.26, .65)*** | .19 (-.02, .40) | .39 (.18, .59)*** |
|  | FAQ-I 10-17 | ref | ref | ref |
|  | FAQ-I 18-25 | -.43 (-.56, -.29)*** | -.28 (-.42, -.13)*** | -.40 (-.54, -.26)*** |
|  | FAQ-I 26-33 | -.76 (-.92, -.61)*** | -.55 (-.72, -.38)*** | -.69 (-.85, -.52)*** |
| Model 4  Adjusted for age, sex, diagnosis, education, ACE-III, and GDS-10 | FAQ-I 0 | 1.18 (.87, 1.49)*** | .92 (.59, 1.26)*** | .79 (.47, 1.11)*** |
|  | FAQ-I 1-5 | .74 (.54, .94)*** | .38 (.17, .59)*** | .59 (.39, .79)*** |
|  | FAQ-I 6-9 | .44 (.25, .63)*** | .17 (-.03, .38) | .36 (.17, .56)*** |
|  | FAQ-I 10-17 | ref | ref | ref |
|  | FAQ-I 18-25 | -.41 (-.54, -.29)*** | -.26 (-.40, -.13)*** | -.39 (-.51, -.26)*** |
|  | FAQ-I 26-33 | -.73 (-.88, -.58)*** | -.51 (-.68, -.35)*** | -.64 (-.80, -.49)*** |
| Model 5  Adjusted for age, sex, diagnosis, education, ACE-III, GDS-10, and RSS | FAQ-I 0 | .80 (.51, 1.09)*** | .65 (.32, .98)*** | .47 (.16, .77)** |
|  | FAQ-I 1-5 | .44 (.25, .62)*** | .17 (-.04, .38) | .34 (.14, .53)*** |
|  | FAQ-I 6-9 | .30 (.13, .48)*** | .09 (-.12, .28) | .25 (.07, .43)** |
|  | FAQ-I 10-17 | ref | ref | ref |
|  | FAQ-I 18-25 | -.25 (-.36, -.13)*** | -.14 (-.28, -.01)* | -.24 (-.37, -.12)*** |
|  | FAQ-I 26-33 | -.42 (-.57, -.28)*** | -.31 (-.46, -.14)*** | -.39 (-.54, -.23)*** |

Note: Addenbrooke’s Cognitive Examination-III, ACE-III; Functional Activities Questionnaire, FAQ; Geriatric Depression Scale-10, GDS-10; Quality of Life in Alzheimer’s Disease, QoL-AD; Satisfaction with Life Scale, SwLS; WHO-5, World Health Organization-Five Well-being Index. * *p*≤.05, ** *p*≤.01, *** *p*≤.001
